# Objective and subjective cognition in survivors of COVID-19 one year after ICU discharge: the role of demographic, clinical, and emotional factors

**DOI:** 10.1186/s13054-023-04478-7

**Published:** 2023-05-15

**Authors:** Marta Godoy-González, Guillem Navarra-Ventura, Gemma Gomà, Candelaria de Haro, Cristina Espinal, Cristina Fortià, Natalia Ridao, Nuria Miguel Rebanal, Laia Oliveras-Furriols, Carles Subirà, Mercè Jodar, Verónica Santos-Pulpón, Leonardo Sarlabous, Rafael Fernández, Ana Ochagavía, Lluís Blanch, Oriol Roca, Josefina López-Aguilar, Sol Fernández-Gonzalo

**Affiliations:** 1 Critical Care Department, Parc Taulí Hospital Universitari, Institut d’Investigació i Innovació Parc Taulí (I3PT-CERCA), Universitat Autònoma de Barcelona, Sabadell, Spain; 2 Department of Clinical and Health Psychology, Universitat Autònoma de Barcelona, Bellaterra, Spain; 3 Centro de Investigación Biomédica en Red de Enfermedades Respiratorias, Instituto de Salud Carlos III, Madrid, Spain; 4 Physical and Rehabilitation Medicine Department, Parc Taulí Hospital Universitari, Institut d’Investigació i Innovació Parc Taulí (I3PT-CERCA), Universitat Autònoma de Barcelona, Sabadell, Spain; 5 Clinical Psychology and Psychobiology Department, Universitat de Barcelona, Barcelona, Spain; 6 Critical Care Department, Althaia Xarxa Assistencial Universitària de Manresa, IRIS Research Institute, Manresa, Spain; 7 Neurology Department, Parc Taulí Hospital Universitari, Institut d’Investigació i Innovació Parc Taulí (I3PT-CERCA), Universitat Autònoma de Barcelona, Sabadell, Spain; 8 Centro de Investigación Biomédica en Red de Salud Mental, Instituto de Salud Carlos III, Madrid, Spain; 9 Medicine Department, Universitat Autònoma de Barcelona, Bellaterra, Spain

**Keywords:** Intensive care, PICS, Cognition, Cognitive reserve, Post-traumatic stress

## Abstract

**Background:**

Intensive Care Unit (ICU) COVID-19 survivors may present long-term cognitive and emotional difficulties after hospital discharge. This study aims to characterize the neuropsychological dysfunction of COVID-19 survivors 12 months after ICU discharge, and to study whether the use of a measure of perceived cognitive deficit allows the detection of objective cognitive impairment. We also explore the relationship between demographic, clinical and emotional factors, and both objective and subjective cognitive deficits.

**Methods:**

Critically ill COVID-19 survivors from two medical ICUs underwent cognitive and emotional assessment one year after discharge. The perception of cognitive deficit and emotional state was screened through self-rated questionnaires (Perceived Deficits Questionnaire, Hospital Anxiety and Depression Scale and Davidson Trauma Scale), and a comprehensive neuropsychological evaluation was carried out. Demographic and clinical data from ICU admission were collected retrospectively.

**Results:**

Out of eighty participants included in the final analysis, 31.3% were women, 61.3% received mechanical ventilation and the median age of patients was 60.73 years. Objective cognitive impairment was observed in 30% of COVID-19 survivors. The worst performance was detected in executive functions, processing speed and recognition memory. Almost one in three patients manifested cognitive complaints, and 22.5%, 26.3% and 27.5% reported anxiety, depression and post-traumatic stress disorder (PTSD) symptoms, respectively. No significant differences were found in the perception of cognitive deficit between patients with and without objective cognitive impairment. Gender and PTSD symptomatology were significantly associated with perceived cognitive deficit, and cognitive reserve with objective cognitive impairment.

**Conclusions:**

One-third of COVID-19 survivors suffered objective cognitive impairment with a frontal-subcortical dysfunction 12 months after ICU discharge. Emotional disturbances and perceived cognitive deficits were common. Female gender and PTSD symptoms emerged as predictive factors for perceiving worse cognitive performance. Cognitive reserve emerged as a protective factor for objective cognitive functioning.

*Trial registration*: ClinicalTrials.gov Identifier: NCT04422444; June 9, 2021.

**Supplementary Information:**

The online version contains supplementary material available at 10.1186/s13054-023-04478-7.

## Introduction

Cognitive, emotional and physical sequelae are common after ICU discharge and may persist for months or even years [1–4]. This set of symptoms, known as Post Intensive Care Syndrome (PICS), may be new onset or worsen previous deficits [1] and have a significant impact on patients’ functionality and quality of life [5]. Focusing on cognitive dysfunction, previous studies have reported a prevalence ranging from 4 to 62% after critical illness [6]. This large variability could be explained by several factors, such as the heterogeneity of clinical diagnoses, the use of different follow-up intervals and assessment tools, and the way cognitive impairment is conceptualized [6]. Impaired cognitive domains include attention, memory, visuospatial ability, executive functions and processing speed [7–9]. In addition, a recent study exploring the existence of different cognitive phenotypes in ICU survivors described a frontal-subcortical profile of cognitive deficits one month after discharge [10]. Other authors have observed a similar pattern of dysfunction at three and twelve months post-ICU [8, 11]. In critically ill patients, the most frequent risk factors for cognitive impairment include delirium [7, 12–17], severity of illness, prior cognitive deficit [7, 15], length of ICU stay [18], mechanical ventilation (MV) [8, 10, 17, 18], and older age [19]. On the other hand, prior studies have found cognitive reserve to be a protective factor against cognitive dysfunction [10, 17, 20]. The pathophysiological mechanisms leading to impaired cognitive performance are still unclear. However, the involvement of neuroinflammation, structural and functional brain alterations, disruption of the blood–brain barrier and alteration of the brain-lung crosstalk have been proposed [8, 21].

The recent coronavirus 2 disease (COVID-19) pandemic led to a significant increase in the number of patients admitted to the ICU and, with it, a greater awareness of the importance of PICS. In COVID-19 patients, a wide range of symptoms persisting over time have been described, including cognitive and psychological disturbances [22]. In a recent prospective multicentre study, 74.3% of COVID-19 survivors reported physical symptoms one-year after ICU discharge, 26.2% mental health problems, and 16.2% cognitive deficits. In addition, 30.6% of survivors reported symptoms in at least two of these domains and 10.5% in all three [23]. Data on long-term cognitive deficits in survivors of COVID-19 are more limited. Studies to date have found rates of dysfunction in global cognition ranging from 15 to 80% [24], with specific deficits in attention, memory, working memory, verbal fluency, executive functions, and processing speed [17, 24–29]. Moreover, these deficits seem to be accentuated in patients who have presented a severe form of the illness, such as those who have survived an ICU admission [29–31].

Due to the pandemic situation, remote assessments have been introduced for evaluating patients’ impairments after hospital discharge. However, measurement of cognition through telematic methods requires, in most cases, the administration of self-rated questionnaires. This practice has generated some controversy regarding the application of subjective measures for detecting objective cognitive deficit in ICU survivors [32]. While objective cognition (OC) provides information on patients' cognitive functioning and is measured through neuropsychological tests administered by experienced professionals, subjective cognition (SC) refers to patients' self-perception of their own cognitive performance, and is usually measured through self-administered questionnaires. A lack of correlation between OC and SC in ICU survivors has been described [32]. Similar results have recently been observed in COVID-19 patients shortly after critical illness [17, 25]. In these studies, OC was mainly related to patients’ demographic and clinical factors, whereas SC was mainly related to their emotional state [17, 25, 32]. However, the relationship between long-term outcomes of OC and SC in COVID-19 ICU survivors and the risk factors involved has scarcely been explored.

Thus, the main aims of this study were (1) to characterize the profile of objective cognitive deficit in survivors of critical illness due to COVID-19 one year after ICU discharge, (2) to study whether the use of measures of subjective cognitive deficit allows the detection of objective cognitive impairment, and (3) to explore the relationship between demographic, clinical and emotional factors and both objective and subjective cognition.

## Methods

### Study design and participants

**Fig. 1:**
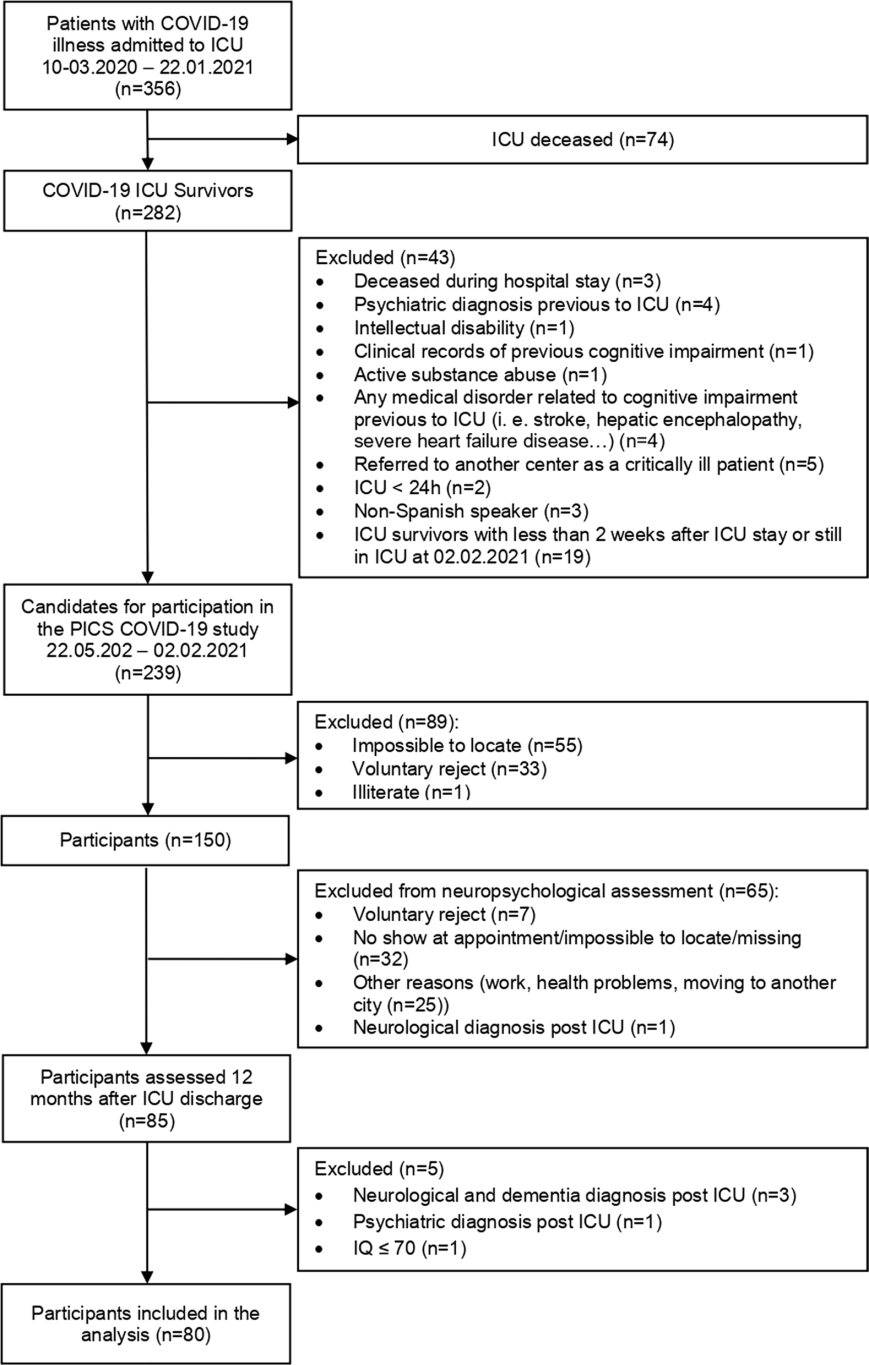
Flowchart representing the distribution of the sample during the different phases of the study

A cross-sectional observational study was proposed to explore long-term objective and subjective cognitive outcomes in COVID-19 ICU survivors, recruited from March 2020 to January 2021. This cohort is part of a larger multicenter study in which survivors of COVID-19 from two medical ICUs in Catalonia, Spain, were followed up for a 12-month period, using a combination of telematic and face-to-face visits (see Fig. 1). The patients included were 18 years of age or older, had a diagnosis of COVID-19 disease with a positive result for SARS-CoV-2, and at least 24 h of ICU admission. Exclusion criteria were: previous neurological disease, severe psychiatric disorder, presence of cognitive impairment or dementia, substance or alcohol abuse, intelligence quotient (IQ) ≤ 70, inability to speak or understand Spanish and non-voluntary acceptance of participation.

The current study was approved by the Institutional Review Board of the Parc Taulí University Hospital (ClinicalTrials.gov Identifier: NCT04422444; June 9, 2021) and is based on an extensive on-site neuropsychological assessment performed one year after ICU discharge (for more details: https://www.tauli.cat/en/institut/projectes-i-xarxes/projectes-financats/pics-covid19/). Informed consent to participate was obtained by telephone after discharge from ICU.

### Data collection

Clinical data were collected retrospectively by reviewing medical records. The presence of delirium was estimated based on the following three criteria: a medical report of delirium, the presence of an episode of agitation, and/or prescription of antipsychotic/neuroleptic treatment during admission. To assess pre-existing cognitive impairment, the relatives of patients ≥ 65 years old completed the Spanish Version of the Short Form of the Informant Questionnaire on Cognitive Decline in the Elderly (Short-IQCODE) [33], which comprises 17 questions to be filled in by a family member or close relative on the cognitive changes perceived in the patient in the last 10 years. A score higher than 57 suggests a possible pre-existing cognitive impairment and was considered an exclusion criterion for participation. Severity of illness was measured by the Acute Physiology and Chronic Health Evaluation (APACHE II) and level of comorbidity by the Charlson Comorbidity Index. Days of mechanical ventilation and length of ICU and hospital stay were also recorded.

### Cognitive and emotional variables

To assess the cognitive and emotional status of survivors of COVID-19 one year after ICU discharge, we administered a full neuropsychological battery to detect OC deficits, a self-reported questionnaire of perceived cognitive deficits to detect SC deficits, and two mental health questionnaires to detect symptoms of anxiety, depression and post-traumatic stress disorder (PTSD). In addition, a questionnaire to assess cognitive reserve was administered after discharge from ICU to evaluate the brain's resistance to a brain insult.

#### Objective cognitive assessment

OC was measured through a comprehensive neuropsychological battery administered by two trained neuropsychologists lasting 45 to 60 min. The battery included the following tests: Digit Span Forward and Backward from the Wechsler Adult Intelligence Scale, 3rd edition (WAIS-III; Wechsler, 1999); Spatial Score Forward and Backward from the Wechsler Memory Scale, 3rd edition (WMS-III; Wechsler, 2004); Rey Auditory Verbal Learning Test (RAVLT); 10/36 Spatial Recall Test (SPART); Stroop Color and Word Test (SCWT); Trail Making Test (TMT) part A and B (for participants with low educational level, we used Color Trails Test, CTT); and phonetic verbal fluency (FAS). To estimate premorbid IQ, we used the Spanish version of the National Adult Reading Test (NART) (for participants with low educational level, we used the Vocabulary subtest of the Wechsler Adult Intelligence Scale, 4th edition, WAIS-IV) (see references in Additional file 1).

For the purposes of this study, raw scores from all cognitive tests were transformed into z-scores (mean = 0; SD =  ± 1) using the normative population data provided for each test, correcting the effects of age and education. Subsequently, seven cognitive indexes were calculated: attention, working memory, learning memory, delayed recall, memory recognition, processing speed and executive functions (see Additional file 2: Table S1). In addition, participants were distributed into two groups, with or without OC impairment, using the classical criterion proposed by Jackson et al. [34]. According to these authors, the presence of objective cognitive deficit occurs when a subject obtains a *z*-score below two standard deviations (SD) in one domain or two *z*-scores below 1.5 SD in two domains. Regarding the level of severity of the cognitive deficit, a moderate cognitive impairment is defined as a *z*-score 1.5 SD below the mean and a severe cognitive impairment as a *z*-score 2 SD below the mean.

#### Subjective cognitive assessment

SC was evaluated using the Perceived Deficits Questionnaire (PDQ), a 20-item questionnaire that assesses different aspects of cognition (attention, memory and executive functions), with a total score ranging from 0 to 68, in which the higher the score, the greater the cognitive deficit perceived by the patient [35]. Participants were categorized into those who perceived the deficit and those who did not, based on the cut-off score of the Spanish validation for clinically significant subjective cognitive deficit (≥ 35) [36].

#### Cognitive reserve

Cognitive reserve was measured using the Cognitive Reserve Questionnaire (CRQ), a questionnaire validated in the Spanish population and consisting of eight items assessing different aspects of the subject’s intellectual activity: level of education, occupation, training courses, language skills, musical training and cognitive stimulating activities, such as reading and intellectual games [37]. The total score ranges from 0 to 25 with higher scores indicating greater cognitive reserve.

#### Emotional assessment

Two mental health questionnaires were used to detect emotional alterations. Anxiety and depressive symptomatology were assessed with the Hospital Anxiety and Depression Scale (HADS) [38], a tool widely used for the detection of psychological symptoms in critically ill patients [39]. It consists of 14 items with two subscales, seven questions for anxiety and seven for depression, with a cut-off score for clinically significant symptoms of ≥ 8 for each subscale. PTSD symptoms were evaluated using the Davidson Trauma Scale (DTS), a well validated questionnaire ranging from 0–136 and comprising 17 items, in which the cut-off score for clinically significant symptoms is ≥ 27 [40].

### Statistical analysis

Analysis was performed using SPSS v.28.0 and statistical significance was established at *p* < 0.05. Data normality was explored with the Kolmogorov–Smirnov test. Descriptive analysis of demographic, clinical, cognitive and emotional variables is presented as medians (range) for continuous variables and percentages (%) for categorical variables. To further characterize the objective cognitive deficit of the survivors of COVID-19, a descriptive graphical analysis with boxplots was used and subsequent analysis of significant differences between cognitive indexes was performed using the Mann–Whitney *U* test. A discriminant analysis based on the Receiver Operating Characteristic (ROC) curve analysis was performed to study the discrimination ability of the PDQ (subjective cognition) to properly categorize subjects with and without objective cognitive impairment. Finally, a series of Mann–Whitney *U* tests and Chi-square tests (*χ*^2^) were used to screen the demographic and clinical variables related to objective and subjective cognitive deficit, as appropriate. Variables found to be significant in these screening analyses were included in two subsequent logistic regression models, one for OC and one for SC. These two logistic regression analyses were performed to identify factors related to objective and subjective cognitive deficits respectively.

## Results

### Demographic and clinical variables

Out of 356 COVID-19 patients admitted during the study period, 150 met the inclusion criteria after discharge. Of these, 85 completed the OC assessment 12 months post-ICU and 75 of them answered the self-administered questionnaires of SC and emotional symptoms. A total of 80 patients were included in the final analysis (Fig. 1), 5 participants were excluded due to psychiatric/neurological disorder diagnosed after recruitment and IQ ≤ 70 assessed on the face-to-face visit. Demographic and clinical characteristics of the sample are shown in Table 1.

**Table 1:** Demographic and clinical characteristics of the sample

Demographic and clinical variables	Total sample (*n* = 80)
Age (years)	60.73 (33.15–79.64)
Female gender	25 (31.3%)
Charlson Comorbidity Index	2 (0–5)
APACHE II score	8 (2–32)
Need for mechanical ventilation	49 (61.3%)
Patients with delirium	33 (41.3%)
Length of ICU stay (days)	11 (2–74)
Length of hospital stay (days)	31.50 (7–107)
Cognitive reserve questionnaire (CRQ)	11 (2–21)
Perceived deficits questionnaire (PDQ)	23 (0–60)
HADS anxiety total score	5 (0–16)
HADS depression total score	3 (0–16)
DTS Total score (PTSD)	13.50 (0–91)

Data are expressed as *n* (%) or median (range), as appropriate

APACHE, Acute physiology and chronic health evaluation; ICU, Intensive care unit; HADS, Hospital Anxiety and Depression Scale; PTSD, post-traumatic stress disorder; DTS, Davidson Trauma Scale

### Cognitive and emotional variables

Out of 80 patients, 30% (*n* = 24) suffered objective cognitive deficits and 27.5% (*n* = 22) reported clinically significant subjective cognitive deficits. Regarding emotional state, 22.5% (*n* = 18) reported mild-to-severe anxiety and 26.3% (*n* = 21) depressive symptomatology, and 27.5% (*n* = 22) reported PTSD symptoms.

### Differences in cognitive performance between groups with and without impaired OC

**Fig. 2:**
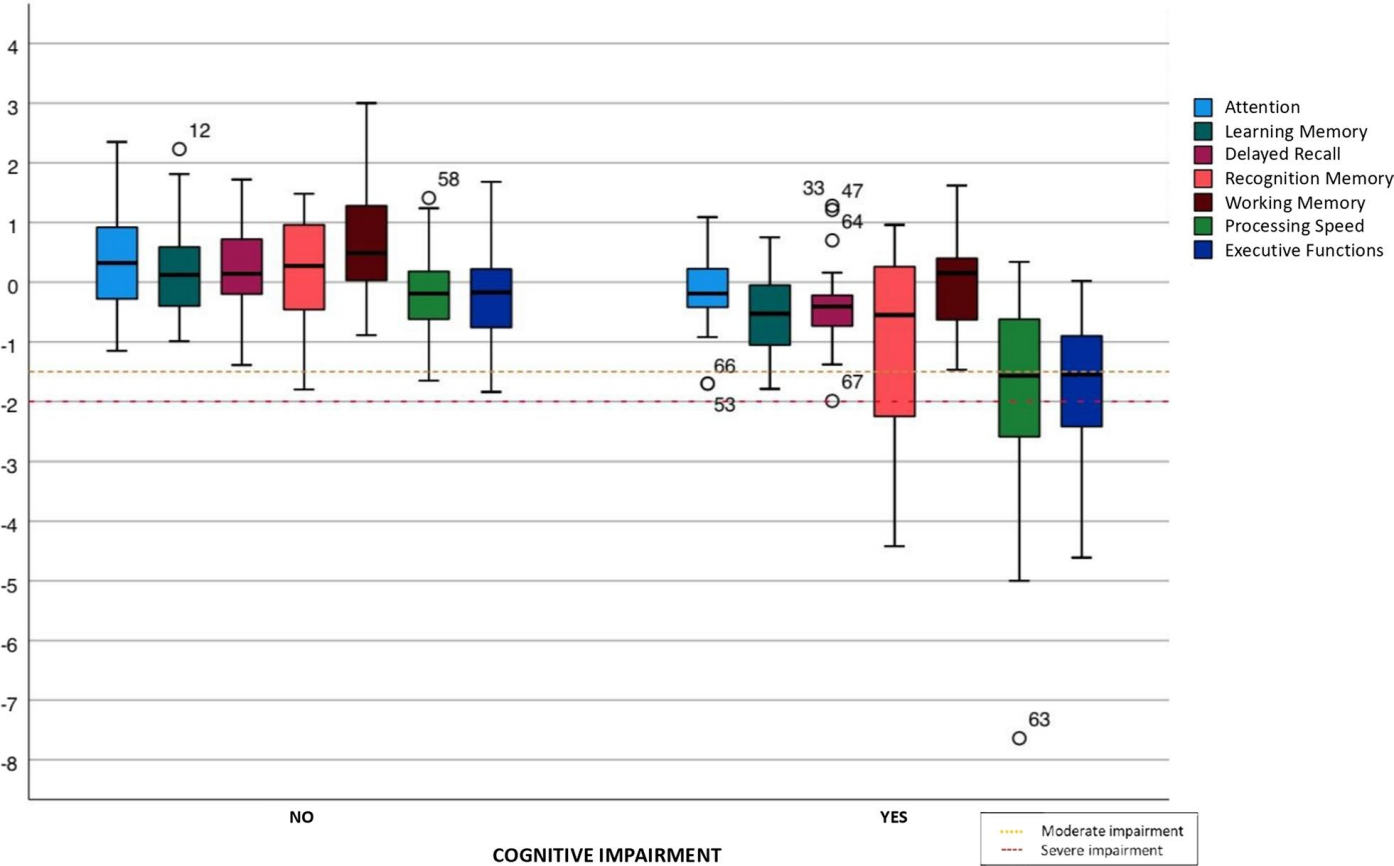
Cognitive profile of patients with and without objective cognitive deficit according to the criteria of Jackson et al. [34]. The seven cognitive indexes are represented in each group. *Z*-scores below − 1.5 standard deviations (SD) are considered as moderate impairment while *z*-scores below − 2 SD are considered as severe impairment

Patients with objective cognitive deficits (according to Jackson’s criteria) showed greater impairment than patients without objective cognitive deficits in executive functions, processing speed, and recognition memory (Fig. 2). Significant differences between the two groups were found in all cognitive indexes (Table 2) and almost all cognitive variables (Additional file 2: Table S2).

**Table 2:** Differences in cognitive performance between groups with and without objective cognitive impairment

Cognitive indexes	Patients without cognitive deficit (*n* = 56)	Patients with cognitive deficit (*n* = 24)	*p* (< 0.05)
Attention	0.31 (− 1.15 to 2.35)	− 0.19 (− 1.70 to 1.09)	0.012
Learning memory	0.14 (− 0.99 to 2.23)	− 0.53 (− 1.79 to 0.75)	< 0.001
Delayed recall	0.14 (− 1.39 to 1.72)	− 0.41(− 1.99 to 1.28)	< 0.001
Recognition memory	0.25 (− 1.80 to 1.48)	− 0.55 (− 4.42 to 0.96)	0.002
Working memory	0.49 (− 0.89 to 3.00)	0.15 (− 1.47 to 1.62)	0.004
Processing speed	− 0.19 (− 1.65 to 1.41)	− 1.57 (− 7.64 to 0.34)	< 0.001
Executive function	− 0.12 (− 1.84 to 1.68)	− 1.55 (− 4.61 to 0.02)	< 0.001

### Correlations and discriminant analysis of OC and SC

**Fig. 3:**
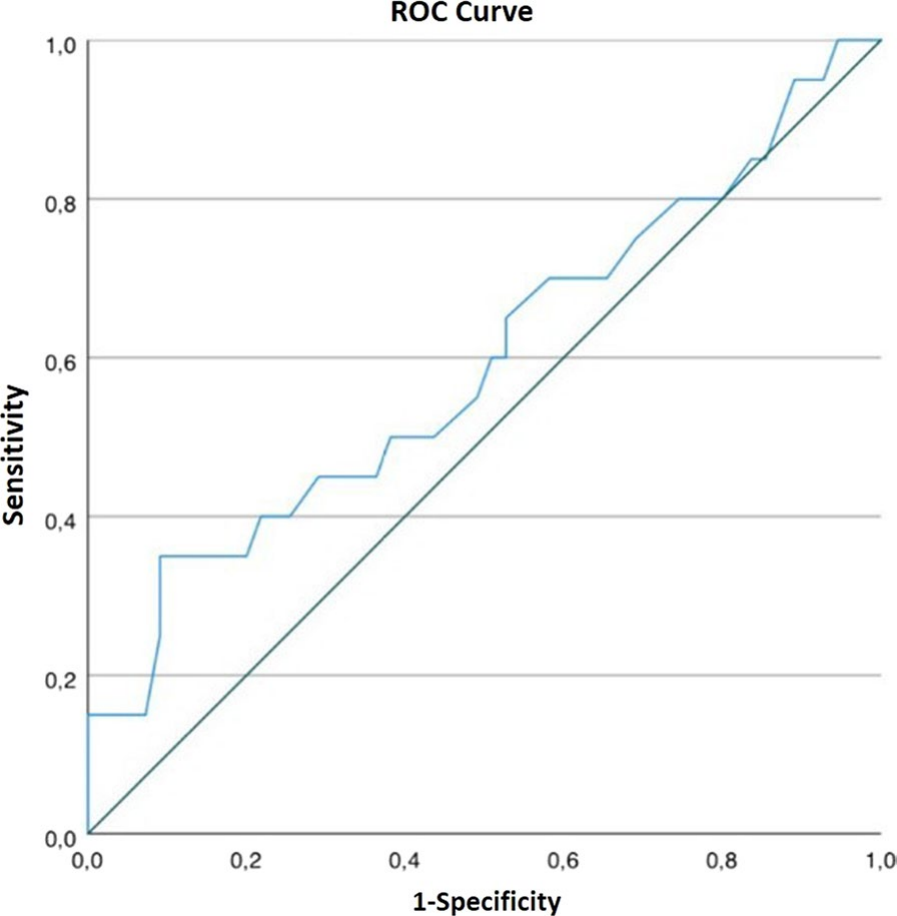
Discriminant analysis of Objective Cognition (OC) and Subjective Cognition (SC): Receiver Operating Characteristics (ROC) curve analysis was performed for the discriminant analysis of OC and SC. The area under the curve (AUC) value was 0.593, indicating that the subjective cognitive measurement (PDQ) did not discriminate well between the groups of patients with and without impaired OC according to the criteria of Jackson et al. [34]

The area under the curve (AUC) value was 0.593, indicating that the PDQ did not discriminate well between the groups of patients with and without impaired OC. The ROC curve analysis of the final discriminant analyses is shown in Fig. 3. Further information about the correlation analysis and the linear regression models for the PDQ subscales and the cognitive indexes are shown in Additional file 2: Tables S3 (Tables S3.1–S3.6).

### Risk factors for OC impairment

The screening analysis of demographic and clinical factors showed that only age and cognitive reserve differed significantly between groups with and without impaired OC, showing that the patients with objective cognitive deficit were older and had lower cognitive reserve than those without this deficit. No significant differences were found between patients with and without impaired OC in perception of cognitive deficit. There were no significant differences in anxiety, depression and PTSD between groups with and without objective cognitive impairment. No other differences were found (further information can be found in Additional file 2: Table S4).

The final logistic regression model explained 10.3% of the variance. Only cognitive reserve reached statistical significance, being inversely related to the impairment in OC (OR = 0.878; 95% CI 0.779–0.988; *p* = 0.031), the greater the cognitive reserve, the lesser the alteration in OC. All other variables, including age, were not significant (Additional file 2: Table S5).

### Risk factors for SC impairment

Significant differences were found in the screening analysis of predicting factors between groups of participants with and without deficits in SC. Participants in the SC deficit group were more likely to be women, were younger and had higher scores in anxiety, depression and PTSD. No other differences were found (further information in Additional file 2: Table S6).

The final logistic regression model explained 38% of the variance. Female gender (OR = 7.650; 95% CI 1.272–46.017; *p* = 0.026), and PTSD symptomatology (OR = 1.105; 95% CI 1.03–1.187; *p* = 0.006) emerged as potential predictors of perception of cognitive deficit. All other variables, including depression and anxiety symptoms, were not significant (Additional file 2: Table S7).

## Discussion

In the current study, one out of three critically ill patients with COVID-19 suffered deficits in OC one year after ICU discharge. Moreover, about 30% of the sample also showed clinically significant difficulties in SC. However, a worse perception of cognitive status, self-assessed by the patient (SC) using the PDQ, did not necessarily correspond with worse outcomes in OC assessed by a professional using an extensive neuropsychological battery. Female gender and symptoms of PTSD were the factors most strongly related to the perception of cognitive deficit, while cognitive reserve emerged as a protective factor against objective cognitive deficit.

The results regarding the prevalence of cognitive dysfunction in non-COVID-19 ICU survivors in previous literature are heterogenous. While Brück et al. [32] found an incidence of cognitive impairment one year after discharge of 16%, the BRAIN-ICU study, which explores a large cohort of medical patients after ICU discharge, showed that one third of ICU survivors experienced impairments in global cognition and executive functions [12]. A review of acute respiratory distress syndrome (ARDS) survivors found that the prevalence of cognitive impairment ranges from 21 to 78% measured from two months to eight years after ICU discharge, although similar rates to the BRAIN-ICU study and to our cohort were found in studies that specifically included cognitive assessment at one year after ICU discharge [41]. However, the literature on long-term OC in ICU COVID-19 survivors remains scarce and the only prospective longitudinal study in COVID-19 patients with ARDS currently available found that only 16% of participants had cognitive impairment one year after discharge, measured with the Montreal Cognitive Assessment (MoCA) [42]. This variability in the rates of cognitive deficit in ICU survivors could be partly explained by methodological differences, for example, by the variety of tests used. In fact, in a recent systematic review, Honarmand et al. [43], found that the prevalence of OC deficits 12 months after ICU discharge was higher when assessed with extensive neuropsychological batteries (43%) than with screening tests (18%) such as the MoCA test, a circumstance that might explain why our rates of alteration in OC in ICU survivors of COVID-19 were higher than in the Latronico et al. [42] cohort. In addition, our results showed notable deficits in executive functions, processing speed and recognition memory, suggesting a frontal-subcortical cognitive dysfunction. Previous studies have observed this pattern in the short [10] and long-term after ICU discharge [11].

On the other hand, 27.5% of our sample manifested clinically significant deficits on the SC questionnaires. Similar results were found by Brück et al. [32], who reported perceived cognitive deficit in 31.6% of non-COVID-19 patients 12 months after ICU discharge. In contrast, Heesakkers et al. [23] found that only 16.2% of COVID-19 survivors reported subjective cognitive symptoms at one year after ICU discharge, although a recent review suggests that the prevalence of SC at long-term follow-up may rise to 45% in this clinical population [43].

Interestingly, we did not observe significant differences in SC between patients with and without OC impairment. Previous studies on critically ill patients with and without COVID-19 have suggested that OC may not necessarily be related to SC [17, 25, 32]. Our results in the discriminant analysis endorse previous findings according to which measures of SC might not adequately detect the long-term OC impairment of ICU survivors. Therefore, and although SC questionnaires have been used before, during, and after the COVID-19 pandemic to screen the level of cognitive deficit in critically ill patients [23, 43], a comprehensive objective assessment of patients' cognitive performance would be necessary to effectively detect cognitive impairment in ICU survivors. These results do not underestimate the relevance of assessing SC in the critical illness population but suggest that it should be considered as a complementary method to the assessment of OC. It should be borne in mind that in our study, emotional symptoms, especially PTSD symptoms, were strongly related to subjective cognitive complaints. This association between poorer scores on SC and emotional symptomatology in ICU survivors recalls those found in the literature in both non-COVID-19 [32, 44] and COVID-19 ICU patients [17, 45].

Regarding emotional state, 22.5% of COVID-19 ICU survivors reported mild to severe symptoms of anxiety, 26.3% depression, and 27.5% PTSD one year after discharge. Prior reviews reported similar [46], or even slightly higher prevalence rates of anxiety symptoms [47] in critically ill survivors. Depressive symptoms can occur in nearly 30% of patients with no pre-existing history of depression at 12–14 months [48], and PTSD symptoms have been reported in 34% of ICU survivors at 1 year follow-up. In COVID-19 post-ICU patients, prevalences of 17.9% for anxiety, 18.3% for depression and 9.8% for PTSD symptoms have been described [49]. Considering the prevalence of emotional alterations in critically ill patients both with and without COVID-19, SC questionnaires may not be the most appropriate measure for estimating objective cognitive status, at least in patients with clinically significant emotional symptoms. Moreover, it should be noted that the assessment took place in a controlled context, while the demands of a more stressful day-to-day environment, for example when returning to work, may have an impact on participants’ cognitive performance. Female gender was also significantly associated with the perception of more severe cognitive deficit, coinciding with the results obtained by an earlier study of a cohort of COVID-19 survivors assessed 6 months after ICU discharge. Although this is beyond the scope of the present paper, further studies might explore whether there are gender differences in emotional symptomatology, as subjective cognitive complaints have been found to be strongly related to psychological symptoms.

Finally, when comparing groups with and without dysfunction in OC, we observed that high cognitive reserve was a predictor of not developing objective cognitive deficit. In previous studies of post-ICU survivors, cognitive reserve has also been identified as a protective factor for patients’ cognitive status [10, 17, 20]. Unlike other authors, we did not find an association between other clinical characteristics and objective cognitive deficits. Delirium is one of the variables most strongly related to long-term objective cognitive impairment [12, 14, 16] and duration of delirium has been linked to worse cognitive performance one year after discharge [12, 16]. In older adults, ICU admission with delirium was linked to greater declines in memory and language [13]. This lack of association might be attributed to the non-prospective collection of data in our study; the presence of delirium was checked retrospectively by reviewing medical records and this information may have been compromised by the fact that it was recorded during the pandemic. In another prospective cohort [50] in which the authors did not find a significant association between delirium and cognitive performance one year after discharge, the results were attributed to the low rates of delirium (36%) compared with those found in Pandharipande et al. [12] cohort. These outcomes are similar to our rates (41%), and could partly explain the lack of association. Another factor that has been linked to cognitive impairment is MV. Previous studies have reported worse objective cognitive functioning in patients with ARDS [18] and in critically ill COVID-19 survivors who underwent MV [17]. Although we found no significant relationship between MV and OC, a recent systematic review 12 of 14 studies also failed to observe a relationship between MV and long-term OC deterioration [16]. In any case, the role of MV in cognitive impairment should continue to be a subject of study.

One of the limitations of the present study, also common in most research in critically ill survivors, is the absence of a baseline neuropsychological examination of patients' cognitive functioning before ICU admission. However, we excluded participants with a diagnosis of dementia/cognitive deficit or neurological diagnosis, and we tried to estimate participants’ prior cognitive state using the Short-IQCODE questionnaire. In addition, we were unable to carry out a baseline cognitive assessment at ICU discharge due to the pandemic context. Another limitation is the lack of a control group of non-critically ill patients with SARS-COV-2 infection and a control group of non-COVID-19 post-ICU patients to assess whether the observed alteration is different in these patient groups. Finally, as the sample size is relatively small, the results of this study should be interpreted with caution.

Future studies should continue to explore the long-term cognitive and emotional sequelae of COVID-19 critically ill patients, observing their evolution over time and detecting possible risk and protective factors. They should use extensive neuropsychological batteries to better characterize their cognitive impairment, in order to provide the best possible intervention in ICU survivors and to prevent these alterations from becoming chronic. Inquiring the long-term functional impact that the perception of cognitive deficit has on critically ill patients may also be of interest.

## Conclusions

This is one of the first studies to explore cognitive function with a complete neuropsychological battery in COVID-19 ICU survivors one year after discharge. Whereas most studies to date have focused on the assessment of global cognition and self-reported cognitive deficit, the results presented here broaden our understanding of specific cognitive domains that are altered. We conclude that one year after discharge one third of COVID-19 post-ICU patients still present impaired OC and around 30% report clinically significant difficulties on SC questionnaires. However, SC questionnaires may not be the most appropriate assessment instruments for detecting deficits in OC in COVID-19 survivors. Only cognitive reserve emerged as a protective factor against OC dysfunction. In contrast, emotional symptoms, especially PTSD, were strongly related to SC. Both OC and SC should be taken into account in the recovery of COVID-19 ICU patients.

## Supplementary Information

**Additional file 1**: References for neuropsychological tests.

**Additional file 2**: **Table S1.** Cognitive indexes, tests used in the neuropsychological assessment battery and formula for calculating each index. **Table S2.** Median z-scores and differences of the cognitive variables between groups with and without objective cognitive deficit. **Table S3.** The correlation analysis and the linear regression models for the PDQ subscales and the cognitive indexes. **Table S4.** Demographic and clinical differences between groups with and without objective cognitive deficit. **Table S5.** Final logistic regression model for objective cognition. **Table S6.** Demographic and clinical differences between groups with and without subjective cognitive deficit. **Table S7.** Final logistic regression model for subjective cognition.

## Data Availability

The datasets used and analyzed during this study are available from the corresponding author on reasonable request.

## References

[1] Needham DM, Davidson J, Cohen H, Hopkins RO, Weinert C, Wunsch H (2012). Improving long-term outcomes after discharge from intensive care unit: report from a stakeholders’ conference. Crit Care Med.

[2] Marra A, Pandharipande PP, Girard TD, Patel MB, Hughes CG, Jackson JC (2018). Co-occurrence of post-intensive care syndrome problems among 406 survivors of critical illness. Crit Care Med.

[3] Inoue S, Hatakeyama J, Kondo Y, Hifumi T, Sakuramoto H, Kawasaki T (2019). Post-intensive care syndrome: its pathophysiology, prevention, and future directions. Acute Med Surg.

[4] Mikkelsen ME, Still M, Anderson BJ, Bienvenu OJ, Brodsky MB, Brummel N (2020). Society of critical care medicine’s international consensus conference on prediction and identification of long-term impairments after critical illness. Crit Care Med.

[5] Kang J, Jeong YJ, Hong J (2021). The effect of postintensive care syndrome on the quality of life of intensive care unit survivors: a secondary analysis. Aust Crit Care.

[6] Wolters AE, Slooter AJC, van der Kooi AW, van Dijk D (2013). Cognitive impairment after intensive care unit admission: a systematic review. Intensive Care Med.

[7] Wilcox ME, Brummel NE, Archer K, Ely EW, Jackson JC, Hopkins RO (2013). Cognitive dysfunction in ICU patients: risk factors, predictors, and rehabilitation interventions. Crit Care Med.

[8] Rengel KF, Hayhurst CJ, Pandharipande PP, Hughes CG (2019). Long-term cognitive and functional impairments after critical illness. Anesth Analg.

[9] Patel MB, Morandi A, Pandharipande PP (2015). What’s new in post-ICU cognitive impairment?. Intensive Care Med.

[10] Fernández-Gonzalo S, Navarra-Ventura G, Bacardit N, Gomà Fernández G, de Haro C, Subirà C (2020). Cognitive phenotypes 1 month after ICU discharge in mechanically ventilated patients: a prospective observational cohort study. Crit Care.

[11] Hughes CG, Patel MB, Jackson JC, Girard TD, Geevarghese SK, Norman BC (2017). Surgery and anesthesia exposure is not a risk factor for cognitive impairment after major noncardiac surgery and critical illness. Ann Surg.

[12] Pandharipande PP, Girard TD, Jackson JC, Morandi A, Thompson JL, Pun BT (2013). Long-term cognitive impairment after critical illness. N Engl J Med.

[13] Schulte PJ, Warner DO, Martin DP, Deljou A, Mielke MM, Knopman DS (2019). Association between critical care admissions and cognitive trajectories in older adults. Crit Care Med.

[14] Girard TD, Jackson JC, Pandharipande PP, Pun BT, Thompson JL, Shintani AK (2010). Delirium as a predictor of long-term cognitive impairment in survivors of critical illness. Crit Care Med.

[15] Müller A, von Hofen-Hohloch J, Mende M, Saur D, Fricke C, Bercker S (2020). Long-term cognitive impairment after ICU treatment: a prospective longitudinal cohort study (Cog-I-CU). Sci Rep.

[16] Sakusic A, O’Horo JC, Dziadzko M, Volha D, Ali R, Singh TD (2018). Potentially modifiable risk factors for long-term cognitive impairment after critical illness: a systematic review. Mayo Clin Proc.

[17] Costas-Carrera A, Sánchez-Rodríguez MM, Cañizares S, Ojeda A, Martín-Villalba I, Primé-Tous M (2022). Neuropsychological functioning in post-ICU patients after severe COVID-19 infection: the role of cognitive reserve. Brain Behavior Immunity Health.

[18] Hopkins RO, Suchyta MR, Snow GL, Jephson A, Weaver LK, Orme JF (2010). Blood glucose dysregulation and cognitive outcome in ARDS survivors. Brain Inj.

[19] Collet MO, Egerod I, Thomsen T, Wetterslev J, Lange T, Ebdrup BH (2021). Risk factors for long-term cognitive impairment in ICU survivors: a multicenter, prospective cohort study. Acta Anaesthesiol Scand.

[20] Navarra-Ventura G, Gomà G, de Haro C, Jodar M, Sarlabous L, Hernando D (2021). Virtual reality-based early neurocognitive stimulation in critically ill patients: a pilot randomized clinical trial. J Pers Med.

[21] Albaiceta GM, Brochard L, Dos Santos CC, Fernández R, Georgopoulos D, Girard T (2021). The central nervous system during lung injury and mechanical ventilation: a narrative review. Br J Anaesth.

[22] Nalbandian A, Sehgal K, Gupta A, Madhavan MV, McGroder C, Stevens JS (2021). Post-acute COVID-19 syndrome. Nat Med.

[23] Heesakkers H, Van Der Hoeven JG, Corsten S, Janssen I, Ewalds E, Simons KS (2022). Clinical outcomes among patients with 1-year survival following intensive care unit treatment for COVID-19. JAMA J Am Med Assoc.

[24] Daroische R, Hemminghyth MS, Eilertsen TH, Breitve MH, Chwiszczuk LJ (2021). Cognitive impairment after COVID-19-A review on objective test data. Front Neurol.

[25] Almeria M, Cejudo JC, Sotoca J, Deus J, Krupinski J (2020). Cognitive profile following COVID-19 infection: clinical predictors leading to neuropsychological impairment. Brain Behav Immun Health.

[26] Mattioli F, Stampatori C, Righetti F, Sala E, Tomasi C, De Palma G (2021). Neurological and cognitive sequelae of Covid-19: a four month follow-up. J Neurol.

[27] Alemanno F, Houdayer E, Parma A, Spina A, Del Forno A, Scatolini A (2021). COVID-19 cognitive deficits after respiratory assistance in the subacute phase: a COVID-rehabilitation unit experience. PLOS ONE.

[28] Jaywant A, Vanderlind WM, Alexopoulos GS, Fridman CB, Perlis RH, Gunning FM (2021). Frequency and profile of objective cognitive deficits in hospitalized patients recovering from COVID-19. Neuropsychopharmacology.

[29] Ollila H, Pihlaja R, Koskinen S, Tuulio-Henriksson A, Salmela V, Tiainen M (2022). Long-term cognitive functioning is impaired in ICU-treated COVID-19 patients: a comprehensive controlled neuropsychological study. Crit Care.

[30] Taquet M, Geddes JR, Husain M, Luciano S, Harrison PJ (2021). 6-month neurological and psychiatric outcomes in 236 379 survivors of COVID-19: a retrospective cohort study using electronic health records. Lancet Psychiatry.

[31] Bailey EK, Steward KA, VandenBussche Jantz AB, Kamper JE, Mahoney EJ, Duchnick JJ (2021). Neuropsychology of COVID-19: anticipated cognitive and mental health outcomes. Neuropsychology.

[32] Brück E, Larsson JW, Lasselin J, Bottai M, Hirvikoski T, Sundman E (2019). Lack of clinically relevant correlation between subjective and objective cognitive function in ICU survivors: a prospective 12-month follow-up study. Crit Care.

[33] Morales González JM, González-Montalvo JI, Del Ser QT, Bermejo PF (1992). Validation of the S-IQCODE: the Spanish version of the informant questionnaire on cognitive decline in the elderly. Arch Neurobiol (Madr).

[34] Jackson JC, Girard TD, Gordon SM, Thompson JL, Shintani AK, Thomason JWW (2010). Long-term cognitive and psychological outcomes in the awakening and breathing controlled trial. Am J Respir Crit Care Med.

[35] Sullivan MJ, Edgley K, Dehoux E (1990). A survey of multiple sclerosis: I. Perceived cognitive problems and compensatory strategy use. Can J Rehabil.

[36] Navarra-Ventura G, Fernandez-Gonzalo S, Serra-Blasco M, Vicent-Gil M, Palao D, Cardoner N (2019). Cognitive failures in healthy middle-aged Spanish adults: a cross-sectional study describing magnitude categories of subjective cognitive deficits. Eur J Psychiatry.

[37] Rami L, Valls-Pedret C, Bartrés-Faz D, Caprile C, Solé-Padullés C, Castellvi M (2011). Cognitive reserve questionnaire. Scores obtained in a healthy elderly population and in one with Alzheimer’s disease. Rev Neurol.

[38] Zigmond AS, Snaith RP (1983). The hospital anxiety and depression scale. Acta Psychiatr Scand.

[39] Bjelland I, Dahl AA, Haug TT, Neckelmann D (2002). The validity of the Hospital Anxiety and Depression Scale: an updated literature review. J Psychosom Res.

[40] Davidson JR, Book SW, Colket JT, Tupler LA, Roth S, David D (1997). Assessment of a new self-rating scale for post-traumatic stress disorder. Psychol Med.

[41] Herridge MS, Moss M, Hough CL, Hopkins RO, Rice TW, Bienvenu OJ (2016). Recovery and outcomes after the acute respiratory distress syndrome (ARDS) in patients and their family caregivers. Intensive Care Med.

[42] Latronico N, Peli E, Calza S, Rodella F, Novelli MP, Cella A (2022). Physical, cognitive and mental health outcomes in 1-year survivors of COVID-19-associated ARDS. Thorax.

[43] Honarmand K, Lalli RS, Priestap F, Chen JL, McIntyre CW, Owen AM (2020). Natural history of cognitive impairment in critical illness survivors a systematic review. Am J Respir Crit Care Med.

[44] Brück E, Schandl A, Bottai M, Sackey P (2018). The impact of sepsis, delirium, and psychological distress on self-rated cognitive function in ICU survivors-a prospective cohort study. J Intensive Care.

[45] Almeria M, Cejudo JC, Sotoca J, Deus J, Krupinski J (2020). Cognitive profile following COVID-19 infection: clinical predictors leading to neuropsychological impairment. Brain Behavior Immunity Health.

[46] LaBuzetta JN, Rosand J, Vranceanu AM (2019). Review: post-intensive care syndrome: unique challenges in the neurointensive care unit. Neurocrit Care.

[47] Nikayin S, Rabiee A, Hashem MD, Huang M, Bienvenu OJ, Turnbull AE (2016). Anxiety symptoms in survivors of critical illness: a systematic review and meta-analysis. Gen Hosp Psychiatry.

[48] Rabiee A, Nikayin S, Hashem MD, Huang M, Dinglas VD, Bienvenu OJ (2016). Depressive symptoms after critical illness: a systematic review and meta-analysis. Crit Care Med.

[49] Parker AM, Sricharoenchai T, Raparla S, Schneck KW, Bienvenu OJ, Needham DM (2015). Posttraumatic stress disorder in critical illness survivors: a metaanalysis. Crit Care Med.

[50] Estrup S, Kjer CKW, Vilhelmsen F, Poulsen LM, Gøgenur I, Mathiesen O (2018). Cognitive function 3 and 12 months after ICU discharge—a prospective cohort study. Crit Care Med.

