# Supplementary material for: Objective and subjective cognition in survivors of COVID-19 one year after ICU discharge: the role of demographic, clinical, and emotional factors
Source: Crit Care. 2023 May 15;27:188. doi: 10.1186/s13054-023-04478-7 (PMC10184095; doi:10.1186/s13054-023-04478-7)
Supplement: Supplementary file 1 — Additional file 1: References for neuropsychological tests. [file 13054_2023_4478_MOESM1_ESM.pdf]

Additional File 1: References for Neuropsychological Tests

- Del Ser T, González-Montalvo JI, Martínez-Espinosa S, et al (1997) Estimation of premorbid intelligence in Spanish people with the Word Accentuation Test and its application to the diagnosis of dementia. Brain Cogn 33:343–356
- Wechsler D (1999) Escala de inteligencia Wechsler para Adultos (WAIS-III manual). TEA. Madrid
- Wechsler D (2004) Wechsler Memory Scale III (WMS-III manual). TEA. Madrid
- Rey A (1964) L'examen clinique en psychologie [The clinical psychological examination]. Universitaire de France, Paris
- Golden C, Freshwatere S (2002) The Stroop Color and Word Test: A Manual for Clinical and Experimental Uses. Stoelting, Chicago
- Reitan R, Wolfson D (1985) The Halstead-Reitan Neuropsychological Test Battery. Clinical Neuropsychological Press, Tucson
- Artiola i Fortuny L, Hermosillo Romo D, Heaton R, et al (1999) Manual de normas y procedimientos para la batería neuropsicológica en español. M Press, Tucson
- Rao, SM. Cognitive Function Study Group, N. A manual for the Brief Repeatable Battery of Neuropsychological Tests in Multiple Sclerosis. New York: National Multiple Sclerosis; 1990.
