# Supplementary material for: Objective and subjective cognition in survivors of COVID-19 one year after ICU discharge: the role of demographic, clinical, and emotional factors
Source: Crit Care. 2023 May 15;27:188. doi: 10.1186/s13054-023-04478-7 (PMC10184095; doi:10.1186/s13054-023-04478-7)
Supplement: Supplementary file 2 — Additional file 2: Table S1. Cognitive indexes, tests used in the neuropsychological assessment battery and formula for calculating each index. Table S2. Median z-scores and differences of the cognitive variables between groups with and without objective cognitive deficit. Table S3. The correlation analysis and the linear regression models for the PDQ subscales and the cognitive indexes. Table S4. Demographic and clinical differences between groups with and without objective cognitive deficit. Table S5. Final logistic regression model for objective cognition. Table S6. Demographic and clinical differences between groups with and without subjective cognitive deficit. Table S7. Final logistic regression model for subjective cognition. [file 13054_2023_4478_MOESM2_ESM.docx]

| **Additional File 2: Table S1.** Cognitive indexes, tests used in the neuropsychological assessment battery and formula for calculating each index. | |
| --- | --- |
| **Attention** | (Digit span forward WAIS-III+ Spatial score forward WMS-III)/2 |
| **Learning memory** | (Number of words learned RAVLT + Number of tokens learned SPART)/2 |
| **Delayed Recall** | (Nº of words recalled in the long term RAVLT + Number of tokens recalled in the long term SPART)/2 |
| **Memory Recognition** | (Nº of words RAVLT recognized in the long term) |
| **Processing speed** | (TMT A + Number of words read in Stroop test)/2 |
| **Working Memory** | (Digit span backwards WAIS-III + Spatial score backwards WMS-III)/2 |
| **Executive Function** | (TMT B - TMT A + Interference score Stroop Test + phonetic verbal fluency, FAS)/3 |
| Abbreviations: RAVLT, Rey Auditory Verbal Learning Test. SPART, Spatial Recall Test. TMT, Trail Making Test. | |

| **Additional file 2: Table S2.** Median z-scores and differences of the cognitive variables between groups with and without objective cognitive deficit. | | | |
| --- | --- | --- | --- |
| ***Cognitive variables*** | ***Participants without objective cognitive***  ***deficit (n=56)*** | ***Participants with***  ***Objective cognitive***  ***deficit (n=24)*** | ***p*** |
| **Attention** |  |  |  |
| DS forward* | 0.155 (-2.09 - 2.37) | -0.55 (-1.63 - 1.57) | 0.009 |
| SS forward | 0.33 (-1.67 - 2.33) | 0.33 (-2 - 1.33) | 0.235 |
| **Working Memory** |  |  |  |
| DS backward* | 0.305 (-0.62 - 2.64) | -0.41 (-1.94 - 1.23) | <0.001 |
| SS backward | 0.67 (-0.67 - 2.67) | 0 (-1 - 2) | 0.075 |
| **Learning Memory** |  |  |  |
| RAVLT Total* | 0.52 (-1.63 - 3.61) | -0.55 (-2.32 - 0.96) | <0.001 |
| SPART Total* | -0.03 (-2.31 - 1.57) | -0.65 (-2.07 - 1.54) | 0.047 |
| **Delayed Recall** |  |  |  |
| RAVLT Recall* | 0.17 (-1.86 - 2.22) | 0.05 (-2.52 - 1.12) | 0.029 |
| SPART Recall* | 0.335 (-1.68 - 2.21) | -0.62 (-1.48 - 1.94) | 0.010 |
| **Recognition Memory** |  |  |  |
| RAVLT Recognition* | 0.58 (-1.80 - 1.48) | -0.64 (-4.42 - 0.96) | 0.002 |
| **Executive Functions** |  |  |  |
| TMT B-A* | -0.65 (-6.79 - 2.15) | -5.09 (-12.74 - -1.35) | <0.001 |
| Stroop Interference | 0.30 (-1 - 1.70) | -0.30 (-1.40 - 2.40) | 0.051 |
| FAS* | 0.035 (-1.91 - 2.59) | -0.66 (-2.23 - 2.20) | 0.007 |
| **Processing Speed** |  |  |  |
| TMT A* | -0.37 (-2.54 - 2.52) | -1.83 (-8.50 - 0.87) | <0.001 |
| Stroop Words* | -0.20 (-2.70 - 1.30) | -0.60 (-2.80 - 0.60) | <0.001 |
| Data are expressed as medians (minimum-maximum)  *p<0.05  Abbreviations: DS, Digit Span; SS, Spatial Score; RAVLT, Rey Auditory Verbal Learning Test; SPART, Spatial Recall Test; TMT, Trail Making Test; FAS, phonetic verbal fluency. | | | |

| **Additional File 2: Tables S3.** The correlation analysis and the linear regression models for the PDQ-20 subscales and the cognitive indexes.  The correlation analysis (Table S3.1) revealed an indirect relationship between several scores of the subjective cognition subscales and the objective cognition indexes. However, although the attention subscale was significantly correlated with the attention index, it was also associated with the working memory index. The retrospective memory subscale correlated significantly with the attention index and working memory, but not with any of the objective memory indexes. The prospective memory subscale was statistically associated with attention, learning memory, delayed recall, working memory, processing speed and executive functions indexes. Finally, the planning-organization subscale correlated with attention, learning memory, working memory, processing speed and executive functions indexes.  Given the multiple correlations of the PDQ subscales with several of the objective cognition indexes five regression models were developed in order to analyze the predictive capacity of the subjective subscales with the objective indexes. The results can be seen in Table S3.2 to S3.6.  Table S3.1. Analysis of correlations between the PDQ subscales and the objective cognition (OC) indexes. 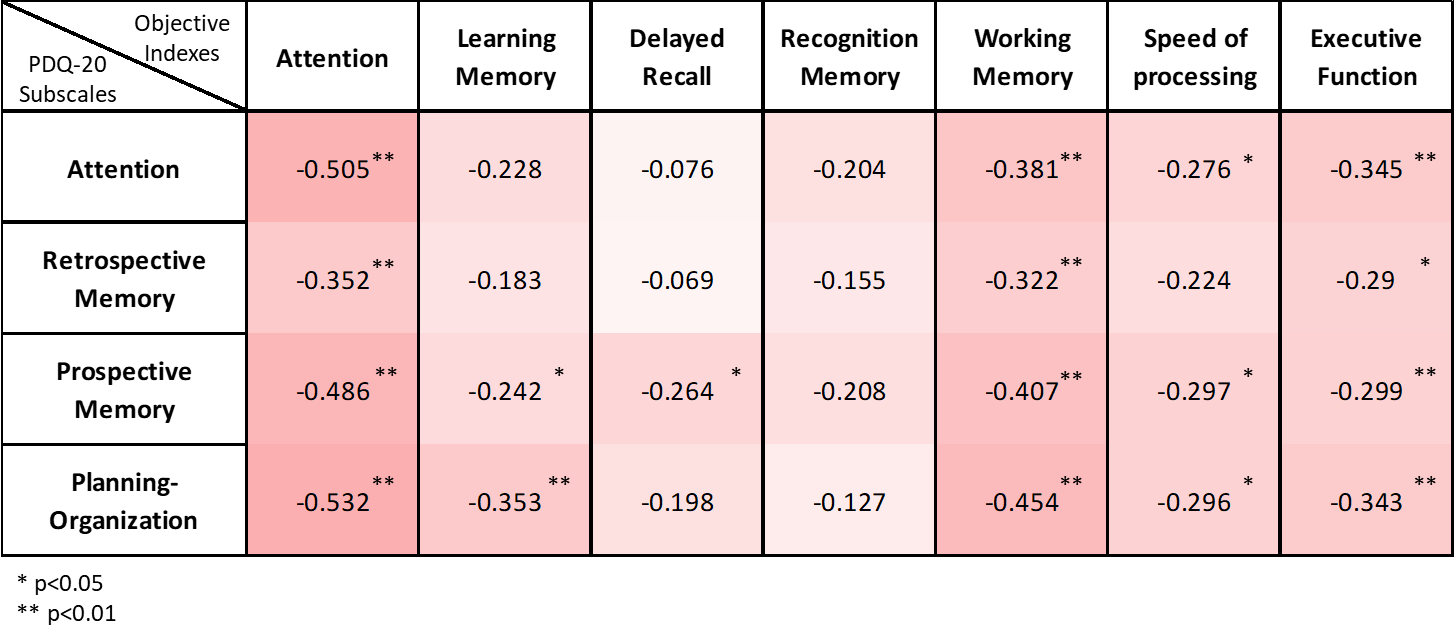 Table S3.2. Linear regression model for predicting Attention Index (R2=0.377)   \|  \| **B** \| **95% CI** \| **β** \| **t** \| **p** \| \| --- \| --- \| --- \| --- \| --- \| --- \| \| Attention \| -0.049 \| [-0.108 , 0.01] \| -0.301 \| -1.653 \| 0.103 \| \| Retrospective Memory* \| 0.092 \| [0.02 , 0.165] \| 0.505 \| 2.537 \| 0.013 \| \| Prospective Memory* \| -0.074 \| [-0.142 , -0.006] \| -0.364 \| -2.179 \| 0.033 \| \| Planning-Organization* \| -0.075 \| [-0.133 , - 0.017] \| -0.433 \| -2.575 \| 0.012 \|   Table S3.3. Linear regression model for predicting Learning Memory Index (R2=0.111)   \|  \| **B** \| **95% CI** \| **β** \| **t** \| **p** \| \| --- \| --- \| --- \| --- \| --- \| --- \| \| Prospective Memory \| 0.008 \| [-0.060 , 0.076] \| -0.037 \| 0.224 \| 0.823 \| \| Planning-Organization* \| -0.063 \| [-0.121 , - 0.005] \| -0.359 \| -2.154 \| 0.035 \|   Table S3.4. Linear regression model for predicting Working Memory Index (R2=0.253)   \|  \| **B** \| **95% CI** \| **β** \| **t** \| **p** \| \| --- \| --- \| --- \| --- \| --- \| --- \| \| Attention \| -0.027 \| [-0.098 , 0.045] \| -0.148 \| -0.744 \| 0.460 \| \| Retrospective Memory \| 0.061 \| [-0.027 , 0.149] \| 0.301 \| 1.381 \| 0.172 \| \| Prospective Memory \| -0.058 \| [-0.140 , -0.024] \| -0.259 \| -1.417 \| 0.161 \| \| Planning-Organization* \| -0.076 \| [-0.147 , -0.006] \| -0.397 \| -2.159 \| 0.034 \|   Table S3.5. Linear regression model for predicting Processing Speed Index (R2=0.146)   \|  \| **B** \| **95% CI** \| **β** \| **t** \| **p** \| \| --- \| --- \| --- \| --- \| --- \| --- \| \| Attention \| -0.068 \| [-0.177 , 0.042] \| -0.241 \| -1.238 \| 0.220 \| \| Prospective Memory \| 0.018 \| [-0.114 , 0.151] \| 0.051 \| 0.276 \| 0.783 \| \| Planning-Organization \| -0.063 \| [-0.178 , 0.052] \| -0.206 \| -1.087 \| 0.281 \|   Table S3.6. Linear regression model for predicting Executive Functions Index (R2=0.181)   \|  \| **B** \| **95% CI** \| **β** \| **t** \| **p** \| \| --- \| --- \| --- \| --- \| --- \| --- \| \| Attention \| -0.057 \| [-0.234 , 0.011] \| -0.236 \| -1.130 \| 0.262 \| \| Retrospective Memory \| 0.012 \| [-0.113 , 0.137] \| 0.044 \| 0.191 \| 0.849 \| \| Prospective Memory \| -0.013 \| [-0.129 , 0.103] \| -0.043 \| -0.222 \| 0.825 \| \| Planning-Organization \| -0.057 \| [-0.157 , 0.043] \| -0.220 \| -1.141 \| 0.258 \| | | | | |  |
| --- | --- | --- | --- | --- | --- | --- | --- | --- | --- | --- | --- | --- | --- | --- | --- | --- | --- | --- | --- | --- | --- | --- | --- | --- | --- | --- | --- | --- | --- | --- | --- | --- | --- | --- | --- | --- | --- | --- | --- | --- | --- | --- | --- | --- | --- | --- | --- | --- | --- | --- | --- | --- | --- | --- | --- | --- | --- | --- | --- | --- | --- | --- | --- | --- | --- | --- | --- | --- | --- | --- | --- | --- | --- | --- | --- | --- | --- | --- | --- | --- | --- | --- | --- | --- | --- | --- | --- | --- | --- | --- | --- | --- | --- | --- | --- | --- | --- | --- | --- | --- | --- | --- | --- | --- | --- | --- | --- | --- | --- | --- | --- | --- | --- | --- | --- | --- | --- | --- | --- | --- | --- | --- | --- | --- | --- | --- | --- | --- | --- | --- | --- | --- | --- | --- | --- | --- | --- |
| **Additional File 2: Table S4.** Demographic and clinical differences between groups with and without objective cognitive deficit. | | | | | |
|  | ***Patients without objective cognitive deficit (n=56)*** | ***Patients with objective cognitive deficit (n=24)*** | ***U/* χ²** | ***p*** | |
| Age^a,*^ | 58.63 (33.15-79.64) | 67.49 (39.48-78.65) | 480.0 | 0.044 | |
| Female gender^b^ | 15 (26.8%) | 10 (41.7%) | 1.732 | 0.188 | |
| Charlson Comorbidity Index^a^ | 2 (0-4) | 2 (0-5) | 504.5 | 0.072 | |
| APACHE II^a^ | 8 (2-32) | 9 (2-20) | 549.5 | 0.197 | |
| Need for mechanical ventilation^b^ | 37 (66.1%) | 12 (50%) | 1.828 | 0.176 | |
| Patients with delirium^b^ | 23 (41.1%) | 10 (41.7%) | 0.002 | 0.960 | |
| Length of ICU stay^a^ | 11.50 (2-74) | 8.50 (3-38) | 574.0 | 0.303 | |
| Length of hospital stay^a^ | 31.50 (7-97) | 32 (10-107) | 631.5 | 0.671 | |
| CRQ^a,*^ | 12 (2-21) | 9 (4-18) | 435.5 | 0.013 | |
| PDQ^b^ | 14 (25.5%) | 8 (40%) | 1.497 | 0.221 | |
| HADS Anxiety Total score^a^ | 5 (0-14) | 6 (0-16) | 435.5 | 0.201 | |
| HADS Depression Total score^a^ | 2.50 (0-13) | 3 (0-16) | 412.5 | 0.117 | |
| DTS Total score (PTSD)^a^ | 14.50 (0-87) | 11.50 (2-91) | 501.0 | 0.635 | |
| Data are expressed as n (%) or median (minimum-maximum), as appropriate.   1. Mann-Whitney U test | | | | | |
| 1. Chi-Square test (χ²)   * p<0.05 | | | | | |

Abbreviations: APACHE, Acute Physiology and Chronic Health Evaluation; ICU, Intensive Care Unit; CRQ, Cognitive Reserve Questionnaire; PDQ, Perceived Deficits Questionnaire; HADS, Hospital Anxiety and Depression Scale; DTS, Davidson Trauma Scale; PTSD, Post-Traumatic Stress Disorder.

**Additional File 2: Table S5.** Final logistic regression model (Risk factors for OC deficit).

| **Factors** | **OR** | **95% CI** | **SE** | **p** |
| --- | --- | --- | --- | --- |
| **Age** | 1.033 | 0.982 – 1.087 | 0.026 | 0.206 |
| **Cognitive reserve** | 0.878 | 0.779 – 0.988 | 0.061 | 0.031 |

Abbreviations: OR, Odd Ratio; CI, Confidence Interval; SE, Standard Error.

*p<0.05

| **Additional File 2: Table S6.** Demographic and clinical differences between groups with and without subjective cognitive deficit. | | | | |
| --- | --- | --- | --- | --- |
|  | ***Patients without subjective cognitive deficit (n=53)*** | ***Patients with subjective cognitive deficit (n=22)*** | ***U/* χ²** | ***p*** |
| Age^a,*^ | 66.29 (36.97-79.64) | 58.39 (33.15-69.83) | 360.0 | 0.009 |
| Female gender^b,*^ | 12 (22.6%) | 11 (50%) | 5.437 | 0.019 |
| Charlson Comorbidity Index^a^ | 2 (0-5) | 1.50 (0-3) | 429.0 | 0.067 |
| APACHE II^a^ | 9 (3-32) | 7.50 (2-18) | 438.0 | 0.090 |
| Need for mechanical ventilation^b^ | 34 (64.2%) | 12 (54.5%) | 0.605 | 0.437 |
| Patients with delirium^b^ | 24 (45.3%) | 8 (36.4%) | 0.506 | 0.477 |
| Length of ICU stay (days)^a^ | 12 (2-74) | 8 (3-57) | 495.0 | 0.305 |
| Length of hospital stay (days)^a^ | 35 (9-97) | 21.50 (7-107) | 434.0 | 0.083 |
| CRQ^a^ | 11 (2-21) | 10.50 (5-21) | 505.5 | 0.366 |
| PDQ^b^ | 12 (22.6%) | 8 (36.4%) | 1.497 | 0.221 |
| HADS Anxiety Total score^a,**^ | 5 (0-13) | 8.50 (1-16) | 246.5 | <0.001 |
| HADS Depression Total score^a,**^ | 1 (0-13) | 8.50 (1-16) | 219.5 | <0.001 |
| DTS Total score (PTSD)^a,**^ | 10 (0-58) | 44 (6-91) | 147.0 | <0.001 |
| Data are expressed as n (%) or median (minimum-maximum), as appropriate.   1. Mann-Whitney U test | | | | |
| 1. Chi-Square test (χ²)   *p<0.05  **p<0.001 | | | | |
| Abbreviations: APACHE, Acute Physiology and Chronic Health Evaluation; ICU, Intensive Care Unit; CRQ, Cognitive Reserve Questionnaire; PDQ, Perceived Deficits Questionnaire; HADS, Hospital Anxiety and Depression Scale; DTS, Davidson Trauma Scale; PTSD, Post-Traumatic Stress Disorder. | | | | |

**Additional File 2: Table S7.** Final logistic regression model (Risk factors for SC deficit).

| **Factors** | **OR** | **95% CI** | **SE** | **p** |
| --- | --- | --- | --- | --- |
| **Age** | 0.931 | 0.857 – 1.010 | 0.042 | 0.087 |
| **Female gender** | 7.650 | 1.272 – 46.017 | 0.916 | 0.026 |
| **Anxiety** | 0.850 | 0.615 – 1.174 | 0.971 | 0.324 |
| **Depression** | 0.983 | 0.770 – 1.255 | 0.019 | 0.890 |
| **PTSD** | 1.105 | 1.030 – 1.187 | 7.633 | 0.006 |

Abbreviations: OR, Odd Ratio; CI, Confidence Interval; SE, Standard Error; PTSD, Post-Traumatic Stress Disorder.

*p<0.05
